# Genetic Diversity of Type 3 Secretion System in *Burkholderia s.l.* and Links With Plant Host Adaptation

**DOI:** 10.3389/fmicb.2021.761215

**Published:** 2021-10-20

**Authors:** Adrian Wallner, Lionel Moulin, Nicolas Busset, Isabelle Rimbault, Gilles Béna

**Affiliations:** PHIM Plant Health Institute, Université Montpellier, IRD, CIRAD, INRAE, Institut Agro, Montpellier, France

**Keywords:** type three secretion system (T3SS), *Oryza*, root colonization, *Paraburkholderia*, PGPR—plant growth-promoting rhizobacteria, pathogen

## Abstract

*Burkholderia sensu lato* species are prominent for their diversity of hosts. The type 3 secretion system (T3SS) is a major mechanism impacting the interactions between bacteria and eukaryotic hosts. Besides the human pathogenic species *Burkholderia pseudomallei* and closely affiliated species, the T3SS has received little attention in this genus as in taxonomically and evolutionary close genera *Paraburkholderia*, *Caballeronia*, *Trinickia*, and *Mycetohabitans*. We proceeded to identify and characterize the diversity of T3SS types using the genomic data from a subset of 145 strains representative of the species diversity found in the *Burkholderia s.l.* group. Through an analysis of their phylogenetic distribution, we identified two new T3SS types with an atypical chromosomal organization and which we propose to name BCI (*Burkholderia* cepacia complex Injectisome) and PSI (*Paraburkholderia* Short Injectisome). BCI is the dominant T3SS type found in *Burkholderia sensu stricto* (*s.s.*) species and PSI is mostly restricted to the *Paraburkholderia* genus. By correlating their distribution with the ecology of their strains of origin, we propose a role in plant interaction for these T3SS types. Experimentally, we demonstrated that a BCI deficient *B. vietnamiensis* LMG10929 mutant was strongly affected in its rice colonization capacity.

## Importance

The type 3 secretion system (T3SS) is a major driver of the interaction between bacteria and their hosts. Bacterial species of the *Burkholderia* genus *sensu lato* (*s.l.*) are prone to engage with a diversity of hosts yet little is known about the underlying mechanisms driving these interactions. The current interest in these bacteria is significant as they include human and plant pathogens but also symbiotic nitrogen fixing bacteria (i.e., rhizobia) and plant growth promoting species. The T3SS of *Burkholderia s.l.* have rarely been investigated theoretically or experimentally. Here we undertook a characterization of the T3SS landscape using genomic sequence data of 145 representative strains of the species diversity described in *Burkholderia s.l*. We identified unexpected T3SS distribution patterns and uncovered the existence of two new T3SS families. Through an experimental approach, we established a role in plant-interaction for one of the novel T3SS family.

## Introduction

The type three secretion system (T3SS) or injectisome, is a specialized and complex macromolecular structure which is embedded in the membranes of Gram-negative bacteria and acts as a protein delivery machine for intra-cellular secretion into eukaryotic cells. T3SS are relatively rare among plant beneficial strains and endophytes and it is generally considered as a marker of pathogenicity (Reinhold-Hurek and Hurek, 2011; Angus et al., 2014). On these grounds, the T3SS was mainly investigated in human, animal, and plant pathogen models including species of *Yersinia*, *Salmonella*, *Shigella*, *Vibrio*, *Chlamydia*, *Pseudomonas, Erwinia, Xanthomonas*, and *Ralstonia* (Coburn et al., 2007). Indeed, the T3SS has proven to be a promising target for therapy as a number of severe pathogens are depending on it for cell invasion and manipulation as well as long term survival inside their host (Deng et al., 2017). Still, the T3SS sparked much interest for its role in rhizobium symbioses as it is involved in a Nod-factor independent symbiotic signalization pathway between the bacteria and the legume host (Okazaki et al., 2016; Teulet et al., 2019). Nod-factor dependent symbionts also frequently possess a T3SS, which influences their host-range (Skorpil et al., 2005; Jiménez-Guerrero et al., 2015).

Species of the *Burkholderia sensu lato* (*s.l.*) group (i.e., *Burkholderia*, *Caballeronia*, *Mycetohabitans*, *Paraburkholderia*, and *Trinickia* genera) are especially diverse regarding interactions with eukaryotic hosts. Indeed, they have been described in close associations with mammals, insects, plants, fungi and amoebae among others, with varying outcomes depending on the bacterial strain and the host involved. Species that are closely related at the genetic level can have radically different impacts on their host, either displaying pathogenic or mutualistic behavior or even engaging in highly specialized nitrogen-fixing symbiosis with legumes (Compant et al., 2008). Both beneficial and virulent strains have been described to possess injectisome-coding genes (Holden et al., 2009; Lackner et al., 2011a; Vander Broek and Stevens, 2017; Dias et al., 2019).

The T3SS coding regulon is clustered within a ca. 22–50 kb region and encompasses more than 20 genes involved in the machinery’s assembly. Despite being highly conserved across genera, the components were assigned unique names depending on the studied bacteria, making comparisons confusing. We will use the widely adopted secretion and cellular translocation (Sct) nomenclature throughout this study (Hueck, 1998). The core structure of the T3SS machinery is composed of sixteen different proteins among which thirteen are highly conserved across all T3SS types i.e., SctC, SctD, SctJ, SctK, SctL, SctN, SctO, SctQ, SctR, SctS, SctT, SctU, SctV. Additionally, the *sct* cluster can englobe genes coding for secreted effector proteins, transcription regulators and chaperones that maintain the secreted proteins folded until they pass the injection rod. The main phenotypical difference between T3SS used for plant invasion (i.e., Hrp-1, Hrp-2, and Rhc) and those used for animal infection (i.e., SPI-1, SPI-2, Ysc, and Cds) lies in their sheath structure. Animal pathogens have an extracellular extension that resembles a short needle of 50–80 nm (Kubori et al., 1998). Plant pathogens have to get through the plant cell wall and thus have a significantly longer extracellular filament termed pilus, reaching a size of several micrometers (Roine et al., 1997).

All T3SS have a common evolutionary origin with the bacterial flagellum. The diversity of injectisomes was broadened recently and 13 different types were recognized with several additional sub-categories (Abby and Rocha, 2012; Hu et al., 2017). In *Burkholderia s.l.* species, most attention was brought to the melioidosis-causing human pathogen *B. pseudomallei* and the closely related but avirulent *B. thailandensis*. *B. pseudomallei* is known to harbor three different T3SS operons in its genome and notably one from the Inv/Mxi-Spa (SPI-1) family. Indeed, this secretion system was described as being fundamental for host invasion and vacuolar escape and several SPI-1 secreted effectors were identified as responsible for these abilities (Stevens et al., 2002, 2003; Burtnick et al., 2008; Bast et al., 2014). The SPI-1 of *B. pseudomallei* displays homology with the secretion system of other human pathogens such as *Salmonella typhimurium* and *Shigella flexneri* (Attree and Attree, 2001). Beyond the SPI-1, *B. pseudomallei* bears two further injectisomes which are specialized for plant interaction and both belong to the Hrp-2 family (Lee et al., 2010). While the Hrp-2 contribute to disease development on tomato plants, they are not adapted for the infection of rice plants (Manivanh et al., 2017). *B. pseudomallei* is mainly found in paddy fields but has indeed never been observed to cause symptoms on rice plants.

This is unlike *B. glumae* and *B. gladioli*, two specialized rice pathogens causing various symptoms from seedling rot to panicle blight (Ura et al., 2006; Zhou-qi et al., 2016). Both bear a single T3SS of the Hrp-2 type. In *B. glumae*, the T3SS is efficient at translocating effectors to plant cells (Sharma et al., 2013). Still, many effectors under control of the central Hrp-2 regulator *hrpB* are secreted by a T2SS (Kang et al., 2008). The T3SS of *B. gladioli* has yet to be investigated for its implication in rice pathogenesis. It is however involved in mycophagy, which is mediated by the injection of a prophage-tail like effector by the T3SS of *B. gladioli* (Swain et al., 2017). Injectisomes have only scarcely been characterized in *Paraburkholderia* species. The Hrp-2 injectisome of *Paraburkholderia terrae* is involved in the attachment to fungal hyphae, thus promoting their co-migration (Yang et al., 2016). The very first evidence of T3SS driven fungi-bacteria symbiosis was demonstrated for the obligate association between *Mycetohabitans rhizoxinica* and *Rhizopus microsporus*. In the absence of its T3SS, *M. rhizoxinica* was unable to induce the sporulation of its host (Lackner et al., 2011b).

In this study, we took advantage of the increasing amount and quality of publicly available genomic data to conduct the identification and characterization of the T3SS across the genera of the *Burkholderia s.l.* group, and search for correlations with bacterial ecologies and host-interactions. In the process, we identified two novel T3SS types which are exclusively present in *Burkholderia s.l.* species. We further used the rice growth promotion models *Paraburkholderia kururiensis* M130 and *Burkholderia vietnamiensis* LMG10929, bearer of an Hrp-2 and a novel T3SS, respectively, to conduct a preliminary characterization of its role in the interaction with rice.

## Materials and Methods

### Type 3 Secretion System Homology Screening in *Burkholderia s.l.*

We used the SctC, SctN, SctT, and SctV sequences of seven well characterized T3SS representatives (Abby and Rocha, 2012) to identify the T3SS clusters of *Burkholderia s.l.* species through homology screening using BLASTp (Supplementary Table 1). The amino acid sequences of SctC, SctN, SctT, and SctV from the representatives of each T3SS type were individually screened for in a database representing the diversity of *Burkholderia s.l.* with one representative strain for each species (mostly the type strain and alternatively the strain with the genome of highest quality; Supplementary Table 2). The database additionally holds 10 new strains that were isolated from rice roots and belong to either *Burkholderia* or *Paraburkholderia* (Bioproject PRJEB31911 and PRJEB42536). Strains were collected, and genomes sequenced as described in Wallner et al. (2019). Genome annotation was performed by the MicroScope platform (Vallenet et al., 2020). Duplicate hits from different BLAST runs were curated and the hit of highest homology conserved.

### Phylogenetic Reconstructions

To infer the phylogenetic evolution of *Burkholderia s.l.* strains nine coding sequences (*dnaG*, *ftsZ*, *glnA*, *gyrB*, *pykA*, *recA*, *rho*, *rpoB*, and *secA*) of 146 *Burkholderia s. l*. strains and two outgroup strains (i.e., *Ralstonia solanacearum* K60 and *Cupriavidus metallidurans* CH34) were used. The genes were aligned separately using the MUSCLE software and subsequently concatenated resulting in a 16,630 bp alignment. An independent alignment was generated using the same loci for the 80 T3SS-bearing *Burkholderia s.l.* strains.

To specifically study the evolution dynamics of the T3SS, four protein sequences (SctC, SctN, SctT and SctV) were aligned separately using MUSCLE and subsequently concatenated to form a 1,652 aa alignment for 136 T3SS found in *Burkholderia s.l.* and 15 reference strains (Supplementary Table 2). Additional sequences of the protein homologs in the flagella coding cluster (FliI, FliR, FlhA) of *E. coli* were added (No SctC homolog exists) to form the outgroup.

All phylogenetic trees were constructed using the BEAST v1.10.2 software with GTR + G + I and LG + G + I substitution models for nucleotide and amino acid sequences, respectively (as determined to be the most suitable using the MEGA-X built-in tool for model selection), and assuming constant lineage birth rate for each branch with a Yule tree prior (best suited for describing the relationships between individuals from different species). The analysis was run for 5,000,000 generations, sampling every 1,000 generations, and a burn-in of 500,000 was applied. Posterior probabilities above 95% are reported in the form of black dots at the nodes.

### Screening for Promoter Regions and Effectors

The PIP-box promoter region was screened for using the prosite format sequence: T-T-C-G-[CGT]-N(15)-T-T-C-G-[CGT]-N(30,32)-[CT]-A-N(3)-T and the EMBOSS v6.5.7.0 fuzznuc software to infer abundance in target genomes. To specifically detect PIP-box regions that are concurrently found in promoting regions of genes, the EDGAR2.3 platform was used (Blom et al., 2016). Screening for homologs of experimentally validated effectors was conducted using BLASTp with a threshold of 30% alignment identity and 1^–10^ *E*-value, on a database of 515 T3SS effectors involved in pathogenesis or symbiosis (Hu et al., 2017). The 1,538 hits were analyzed with the EffectiveT3 prediction tool (Eichinger et al., 2016) resulting in 464 putative effectors with a prediction score > 0.1. The list was further refined by removing hits in strains lacking any kind of T3SS type and hits resulting from homology with effectors that yielded homologs with low identity in > 90% of screened *Burkholderia s.l.* strains.

### Bacteria Growth Conditions and Insertional Mutagenesis

*Burkholderia vietnamiensis* LMG10929 (*Bv*) and *Paraburkholderia kururiensis* M130 (*Pk*) were cultured using Luria’s low salt LB (LBm; Sigma-Aldrich). Fragments of *Bv*’s *hrcV* and *Pk*’s *hrcC* genes were inserted into a pGEM-T Easy vector (Promega) using specific primers (*hrcV*-fwd: 5′-GTTCATCGTGTCGGTGGTC-3′, *hrcV*-rev: 5′-GAACGACCATTTCGGGAAG-3′, *hrcC*-fwd: 5′-GTTGAAGGCAAAGATCTCAAGG-3′, *hrcC*-rev: 5′-CGCCACCATTACCACCTC-3′) and cloned into heat-shock competent *E. coli* JM109 cells. Positive cells were multiplied, and their plasmid extracted using the Wizard Minipreps DNA purification system (Promega). The gene fragments were liberated from the plasmid by an *Eco*RI digestion and separated on a 1% agarose gel followed by a purification using a NucleoSPin Gel and PCR Clean-up kit (Macherey-Nagel). The target vector pSHAFT2 (Shastri et al., 2017) carrying a chloramphenicol (Cm) resistance gene was opened by an *Eco*RI digestion and treated with an alkaline phosphatase. Insert and plasmid were mixed in a 5:1 ratio regarding their respective molecular size and ligated using a T4 DNA ligase (Promega). The resulting vectors, pSHAFT2-*hrcC* and pSHAFT2-*hrcV*, were cloned into heat-shock competent *E. coli* CC118 (λ*pir*) which were to be used as donor cells during the subsequent triparental mating process (Supplementary Table 3). Spontaneous streptomycin (St) resistant *Bv* and *Pk* mutants were used as acceptor cells. Donor, acceptor (i.e., *Pk*-St^*R*^ and *Bv*-St^*R*^) and *E. coli* DH5α carrying a pRK2013 helper plasmid were grown overnight (in liquid LB at 37°C for *E. coli* species with 50 μg.mL^–1^ kanamycin for helper strain and 50 μg.mL^–1^ chloramphenicol for donor strain; in liquid LBm at 28°C with 100 μmol.L^–1^ streptomycin for *Bv* and *Pk*). The cultures were spun down and the cells were concentrated to OD_600_≈50. Acceptor, donor and helper cells were mixed in a 10:1:1 ratio and 50 μL spots were spread out on square LBm plates. The plates were incubated at 28°C for 12 and 48 h for *Bv* and *Pk*, respectively. The bacterial spots were then resuspended in liquid LBm before being plated on LBm plates supplemented with 100 μg.mL^–1^ streptomycin and chloramphenicol to counter select for the presence of *E. coli* cells and acceptor cells having not integrated the plasmid. To validate the insertion of the plasmid through homologous recombination at the desired T3SS sites, we checked for the positive amplification of the *cat2* Cm-resistance gene and the negative amplification of a set of primers binding upstream and downstream of the targeted area.

### Plant Growth Conditions and Colonization Tests

*Oryza sativa* subsp. *japonica* cv. Nipponbare plants were cultured as described in King et al. (2019). Briefly, seeds were sterilized using successive 70% ethanol and 3.6% sodium hypochlorite treatments and germinated seedlings were transferred onto sterile perlite in an airtight hydroponic system. Plants were grown for 5 days before being inoculated with *Pk*-St^*R*^, *Bv*-St^*R*^, *Pk*-Δ*hrcC*, and *Bv*-Δ*hrcV*. Each individual hydroponic system contained 5 plants. Each plant was inoculated at the stem basis with 1 mL of water containing 1 × 10^8^ bacterial cells. Control plants were inoculated with 1 mL of water. Two systems (2 × 5 plants) were prepared for each condition and time point. At 7 and 14 dpi, the five fittest looking plants were harvested for each condition. The entire root system was sampled and transferred to a screw cap tube containing 1 mL of sterile water and a sterile ceramic bead. Roots were weighted and then pulverized using a Fastprep-24 (MPbio) at 6 m.s^–1^ for 40 s. A serial dilution of the resulting solution was spotted out on square LBm plates supplemented with 100 μg.mL^–1^ streptomycin and incubated at 28°C for 48 h before counting CFU.

## Results

### Phylogenetic Analysis Suggests Two Novel Type 3 Secretion System Types in *Burkholderia s.l.*

**FIGURE 1:**
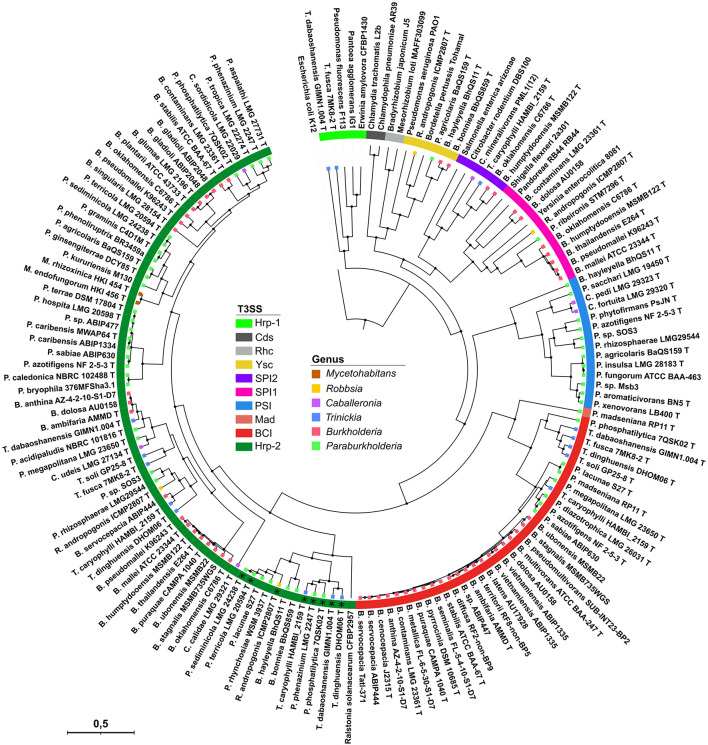
Phylogenetic reconstruction of T3SS cluster evolution amongst *Burkholderia s.l*. species. 80 *Burkholderia s.l.* strains containing a total of 136 secretion systems are represented. Their phylogenetic distribution was inferred using a Bayesian prediction on the concatenated SctC, SctN, SctT, SctV protein sequences resulting in a 1,652 aa alignment. Homologous flagellum components were used for the outgrouping *E. coli* strain in order to root the tree. The colored strip indicates the T3SS types and the colored squares at the branch tips represent the genus of each strain. (*) Eight strains possess a second Hrp-2 injectisome which is phylogenetically close to the one from *Ralstonia solanacearum.*

The screening for injectisome coding regions within 146 *Burkholderia s.l.* strains, representing 139 different species yielded a total of 134 T3SS clusters distributed in 84 strains. Our selection of four core proteins, i.e., SctC, SctN, SctN, SctV, concatenated into a 1652 amino acid alignment and used for the phylogenetic reconstruction resulted in the clustering of *Burkholderia s.l.* T3SS into seven different clades (Figure 1 and Supplementary Table 4). Five of those clades cluster together with reference T3SS which were included to infer homology to validly described T3SS, i.e., Hrp-1, Hrp-2, SPI-1, SPI-2, and Ysc (see Experimental procedures). No *Burkholderia s.l.* T3SS is phylogenetically close to the Rhizobiales- or Chlamydiales-like T3SS. However, two additional categories distinctly appear which do not cluster with any reference sequence (named BCI and PSI, described in detail below). One unique T3SS, belonging to *Paraburkholderia madseniana* RP11 is phylogenetically isolated (Figure 1) and could potentially represent an eighth T3SS category.

**FIGURE 2:**
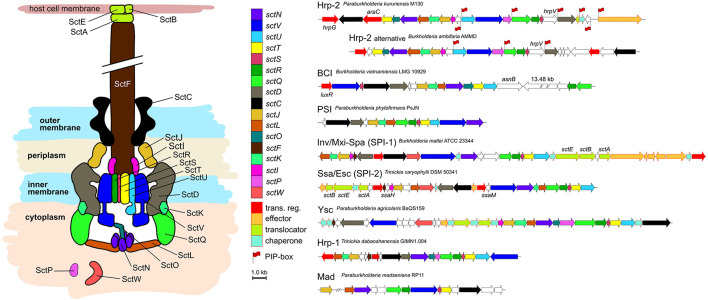
Schematic overview of the T3SS and synteny of coding regions Burkholderia s.l. species. Seven different major types of injectisomes are found in Burkholderia s.l. species. They can be distinguished by their genetic proximity but also by the chromosomic organization of their coding region as depicted here. These regions vary in size and composition while a subset of 12–13 core components is ubiquitously present (*sctO* is absent in PSI, SPI-1, and SPI2; *sctK* is absent in Hrp1). Secretion and cellular translocation genes have an individual color code with a black outline. Further genes involved in transcription or coding for effectors, their translocators and chaperones are depicted with a red outline. Genes without sct nomenclature or with unknown functions are left blank. Red flags at gene beginnings indicate the presence of a PIP-box regulation sequence in the region preceding the CDS.

We analyzed conservation levels of T3SS-related operon synteny within and between the different phylogenetic types found amongst *Burkholderia s.l.* strains (Figure 2). The thirteen core components are consistently found in every T3SS type. The overall chromosomal organization of the T3SS coding operons is strongly varying from type to type. Within T3SS types however, there is little variation even between phylogenetically distant strains sharing the same type of injectisomes, e.g., *Ralstonia solanacearum* CFBP2957 and *Paraburkholderia kururiensis* M130 (Supplementary Figure 1). The new clusters identified in the previous section (BCI and PSI) display a distinct chromosomal organization from the remaining T3SS types (Figure 2).

### *Burkholderia* Cepacia Complex Injectisome and *Paraburkholderia* Short Injectisome: Novel Injectisomes With Differential Distribution

**FIGURE 3:**
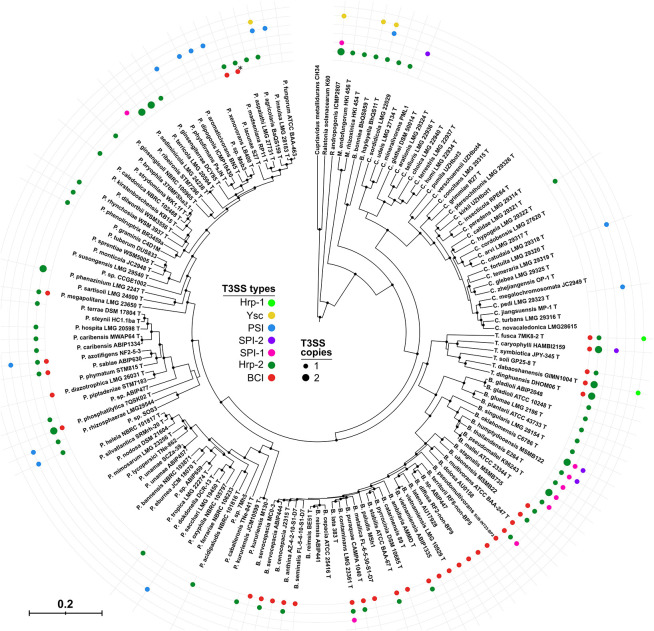
Distribution of T3SS types in *Burkholderia s.l.* species. Nine concatenated coding sequences (*dnaG, ftsZ, glnA, gyrB, pykA, recA, rho, rpoB*, and *secA*) of 146 *Burkholderia s.l.* strains were used to infer the *Burkholderia s.l.* phylogeny using a Bayesian approach. From the outermost circle inwards, colored circles are representative of the presence of an operon coding for the corresponding T3SS. Large Hrp-2 circles indicate the presence of two similar operons in the genome. ^∗^*P. madseniana* displays a peculiar T3SS, Mad, similar to the BCI but phylogenetically distinct.

Based on phylogenetic analysis and operonic organization, we observed two novel injectisomes. The first one, which we refer to as *Paraburkholderia* Short Injectisome (PSI), is dominantly present in *Paraburkholderia* with 11 representative strains. Additionally, two *Caballeronia* species harbor this new type as well as *Burkholderi*a *hayleyella* (Figures 1, 3). Its existence was already reported in studies on *P. phytofirmans* which insisted on its atypical chromosomic organization (Mitter et al., 2013; Angus et al., 2014). Compared to other injectisome families present in *Burkholderia s.l.* the regulon coding for this peculiar T3SS is remarkably short with only 12–14 kb and containing 14–16 genes. Although most structural *sct* components are present, we could not identify *sctF*, the canonical gene coding for the needle-forming component. Still, several genes with unidentified functions within the operon could fulfill this function as they are in the size range of previously reported *sctF* genes. The needle component *sctF* is extremely variable (Lombardi et al., 2019) and is usually undetectable by simple homology approaches. To confirm its presence would require a thorough and dedicated investigation, including protein structure analyses and functional validation (Lombardi et al., 2019).

The second uncharacterized T3SS family, which we refer to as *Burkholderia* cepacia complex Injectisome (BCI), is almost ubiquitously present among species of the *Burkholderia cepacia* complex (BCC). It is further found in four *Trinickia* and seven *Paraburkholderia* species (Figures 1, 3). The operonic structures coding for this injectisome contains the thirteen core genes. A confident identification of the needle component *sctF* proved here again to be unsuccessful. Still, both BCI and PSI types have been transferred independently to multiple species and been conserved throughout speciation (Figure 1). This strongly suggests that the PSI and BCI remain functional and that selection favored their conservation.

The particularity of the BCI is the presence of a large > 10 kb element separating the *sctR* and *sctQ* genes from the rest of the operon (Figure 2). The protein encoded by this large element is predicted to possess a peptidoglycan binding LysM domain. An adjacent gene is encoding a putative lytic transglycolase, allowing peptidoglycan degradation. Another interesting feature is the upstream *luxR*-type regulator which could hint toward a quorum sensing dependent activation of the operon.

By a homology search using the concatenated sequences also used in the T3SS phylogeny, we verified if the PSI and BCI types are present outside *Burkholderia s.l.* species. For the PSI type, the closest hits had a similarity of less than 50% sequence homology and a divergent chromosomal organization. For the BCI, however, there were several hits with > 55% sequence homology and a concordant operon structure. These BCI are mainly found in *Variovorax* species but also in other families of the Burkholderiales order which it seems restricted to Supplementary Figure 2.

The last new T3SS presents no taxonomical closeness to any model used in our phylogenetical reconstruction where it is placed at the interface between the BCI and the PSI (Figure 1). It is found in *P. madseniana* RP11 and also has a chromosomal organization that diverges from the remaining types found in *Burkholderia s.l.* (Figure 2). The available genome for this strain is relatively fragmented (393 contigs) and the T3SS operon is split. Nine out of thirteen core genes could nonetheless be detected and do not show signs of degeneration suggesting that these divergent genes remain active. However, as we do not have any diversity supporting the description of this non-canonical T3SS type we will not further insist on the characterization of this putative injectisome.

### The Injectisome Is a Frequent Feature of *Burkholderia s.l.* Species

Among the *Burkholderia s.l.* representatives tested here, 58% possess a T3SS. However, there are great disparities between genera. Indeed, only six T3SS-positive strains were detected amongst 31 *Caballeronia* species (Figure 3). Several species belonging to this genus were described in endosymbiotic associations with insects or plant leaves (Miller, 1990; Kikuchi et al., 2005) but none are T3SS-positive.

It is not uncommon for *Burkholderia s.l.* species to bear two or more T3SS clusters (22% of strains). For instance, out of the six validly described *Trinickia* species, four have three injectisome-coding clusters of different types.

We attempted to infer correlations between the T3SS type carried by a strain and its ecologic isolation source. The presence of a SPI-1 type is strongly correlated with a strain’s isolation from a clinical setting as it is present essentially in the *Burkholderia mallei* complex (Figure 3 and Supplementary Table 2). While species of the BCC are also frequently described as potential human opportunistic pathogens, they are equally present in association with plants, in the rhizosphere or as environmental strains. The BCI which is the most frequent T3SS among BCC strains, is dominantly found in environmental or plant-associated bacteria (Figure 3 and Supplementary Table 2). The Hrp-2, which has a demonstrated role in plant-interaction, is also occasionally found in clinical species but dominantly in environmental and plant-isolates. Finally, the PSI type T3SS was never detected in clinical isolates among the surveyed *Burkholderia s.l.* strains. Furthermore, 10 out of 16 legume nodulating *Paraburkholderia* and *Trinickia* species lack a T3SS (Figure 3).

The Ysc type injectisome is rare in *Burkholderia s.l.* species (Figure 3). Beside *Robbsia andropogonis* it is restricted to three species which share amoeba as a common host (Haselkorn et al., 2019), *Paraburkholderia agricolaris* and the genetically distant *Burkholderia bonniea* and *B. hayleyella* (Figure 3). Unlike the remaining T3SS types they share, the Ysc appears to be well conserved among these 3 species (Figure 1).

### Injectisomes Have a Complex Evolutionary History in *Burkholderia s.l.* Species

There are great disparities in the distribution of the different T3SS types between different *Burkholderia s.l.* species (Figure 3 and Supplementary Figure 3). The Hrp2 distribution along every genus, and especially in the early diverging genera *Mycetohabitans* and *Robbsia*, suggests that it could have been present in the common ancestor of the *Burkholderia s.l.* genera, subsequently lost in different species and regularly reacquired, supporting its central role in several species. The second most abundant injectisome is the newly described BCI type which is present in almost every species of the BCC and *Trinickia* spp. as well as several *Paraburkholderia* species. The BCI sequences recovered from *Burkholderia s.s.* species form a single clade (Figure 1), suggesting not only a single emergence event in this group, but also its maintenance all along the diversification of this genus, reflecting its evolutionary importance. Both SPI-1 and PSI injectisomes are more occasional and are almost completely restricted to the *Burkholderia mallei* complex (BMC) and a *Paraburkholderia* sub-clade, respectively. The three remaining T3SS types, SPI-2, Hrp-1, and Ysc are only sporadically present in *Burkholderia s.l.* species.

Many *Burkholderia s.l.* species harbor two or more different injectisomes which always belong to different types except for Hrp-2. Eight of the ten species bearing two Hrp-2 have one copy closely related to the Hrp-2 of *R. solanacearum* suggesting the existence of Hrp-2 subfamilies with potential diverging functions.

We further verified if the complex gain and loss dynamics of T3SS are also displayed at the intraspecific level. Many *Burkholderia s.l.* strains were only recently described and are consequently represented by a single whole-genome sequence. Still, several species with either clinical importance or peculiar ecologies and functions (such as nodulation capacity) have received increased attention and had several genomes sequenced.

Using all 87 publicly available genomes, we validated that *B. vietnamiensis* uniformly possesses a single T3SS of the BCI type (screened strains and T3SS types for the following species are listed in Supplementary Table 5). This was likewise verified for all 11 *P. tropica* representatives which uniformly display an Hrp-2 type. *Trinickia symbiotica* is the only representative of its genus lacking a T3SS (Figure 3) and we confirmed that this was consistently valid in all six available strains (Supplementary Table 5). Similarly, the species-wide absence of T3SS was validated for eight *P. caballeronis*, and five *P. silvatlantica* strains. *P. fungorum* is the only representative of PSI-bearing species with more than four representative and sequenced strains. All *P. fungorum* uniformly possess a single T3SS of the PSI type. For all these species, there is therefore a uniformity of their genomic content of secretion systems.

While *B. lata* strain 383 was identified to lack any kind of T3SS, screening of the remaining 23 available genomes of this species yielded a more diverse landscape with eight strains being positive for one T3SS (Supplementary Table 5). Still, the absence of injectisome is the main trend in this species. While not a ubiquitous feature, two *B. servocepacia* strains were detected to have a Hrp-2 injectisome in addition to their BCI. The recently described *B. servocepacia* species englobes strains that were affiliated to *B. cenocepacia* but show significant genomic divergence (Wallner et al., 2019). The Hrp-2 was never observed in *B. cenocepacia* strains despite a wider diversity of available genomes and could thus be a sign of adaptation toward improved interaction with plants that is displayed by *B. servocepacia*.

Our prediction for *B. contaminans* using strain LMG23361, however, seems to be the exception among the diversity of strains for this species. Indeed, the Hrp-2/BCI/SPI-1 trinity of injectisomes was not detected in any other of the 35 genomes available for *B. contaminans*. Only five strains display both an Hrp-2 and a BCI type while the remaining strains present either one of those injectisome types (Supplementary Table 5).

### Effectors of Type 3 Secretion System -Positive “Environmental” Strains Remain Elusive

**FIGURE 4:**
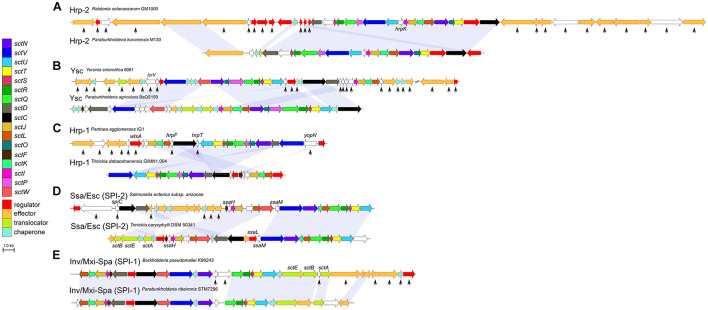
Loss of accessory genes in T3SS synteny blocks of *Burkholderia s.l.* species. The Hrp-2 type **(A)** is the most frequent T3SS in *Burkholderia s.l.* species. The Ysc **(B)**, Hrp-1 **(C)** and SPI-2 **(D)** are T3SS types which rarely occur in *Burkholderia s.l.* species. The SPI-1 type **(E)** is only represented in a single *Paraburkholderia* species. In each case, several genes coding for effectors and chaperones as well as proteins with unknown functions are lost (black arrows) compared to pathogens were these genes have a described role in virulence.

In *Burkholderia s.l.* species, the Hrp-2, Hrp-1, Ysc, and SPI-2 type encoding genetic regions are lacking several genes coding for effectors and chaperones compared to the pathogenic bacteria where these systems have been described (Figures 4A–D). The same trend can be observed for the SPI-1 type of *P. ribeironis* STM7296, a legume nodulating species (Bournaud et al., 2017), which also underwent substantial gene loss compared to other SPI-1 carriers such as *B. pseudomallei* (Figure 4E).

Using a database of 515 validated effectors from animal and plant pathogens but also rhizobia (Hu et al., 2017), we screened for homologies in the genomes of *Burkholderia s.l.* species. We used the effector predictive tool EffectiveT3 to cross-check the resulting list of putative effectors (Eichinger et al., 2016). This approach yielded hits for 62 *Burkholderia s.l.* species (Supplementary Table 6). The species for which we detected the most effectors are the well-studied plant pathogens *T. caryophylli* and *R. andropogonis* (17 and 16 hits, respectively). We also detect the animal pathogens of the *Burkholderia mallei* complex, the plant pathogens of the *Burkholderia glumae* complex and the fungal endosymbionts of the *Mycetohabitans* genus among the species with the most effectors (Supplementary Table 6). Our survey identified several putative effectors in commensal *Paraburkholderia* and *Trinickia* species. Mostly, these candidates share homology with previously validated effectors originating from *Ralstonia*. However, we observed no homology to nodulation enabling Nop effectors amongst the legume nodulating *Paraburkholderia* and *Trinickia* species.

As the Hrp-2 is the dominant T3SS of *Burkholderia s.l.* species, we screened the genomes used in this study for the presence of Plant Inducible Promoter regions (PIP-box; TTCG[CGT]N(15)TTCG[CGT]N(30,32)[CT]AN(3T), indicative of T3SS-linked genetic regulation (Koebnik et al., 2006). Two to eight of these regulatory sequences were found within the operon coding for the T3SS machinery depending on the species (Figure 2). As expected, the total number of PIP-box matching sequences is significantly higher in species possessing an Hrp-2 type injectisome compared to those lacking this type (Wilcoxon test, *p* < 1 × 10^–6^). However, the three polyphyletic Hrp-2 bearing amoeba symbionts (*B. bonniea*, *B. hayleyella* and *P. agricolaris*) are not enriched in PIP-box sequences despite the presence of the PIP-box binding regulator HrpB, suggesting that their Hrp-2 has drifted from its initial function to another purpose.

### Novel *Burkholderia* Cepacia Complex Injectisome Injectisome Is Involved in Rice Root Colonization by *B. vietnamiensis* LMG10929

Two model species of the *Burkholderia* group, *P. kururiensis* strain M130 (*Pk*) and *B. vietnamiensis* strain LMG10929 (*Bv*), are known to form beneficial and intricate relationships with rice. Both heavily colonize the root surface and have been observed as endophytes (Mattos et al., 2008; King et al., 2019). They are potent plant growth promoters which is linked to their ability to transfer fixed atmospheric nitrogen to the plant and to synthetize growth promoting phytohormones (Trân Van et al., 2000; Dias et al., 2019).

*Pk* and *Bv* possess a single injectisome of Hrp-2 and BCI type, respectively (Figure 3). We constructed insertion mutants targeting the *sctC* and *sctV* genes in *Pk* and *Bv*, respectively (Figure 1). The deletion of any of these structural components has repeatedly been used to efficiently inactivate T3 secretion in *Burkholderia s.l.* species (Muangsombut et al., 2008; Lackner et al., 2011a; Gavrilin et al., 2012; Swain et al., 2017).

**FIGURE 5:**
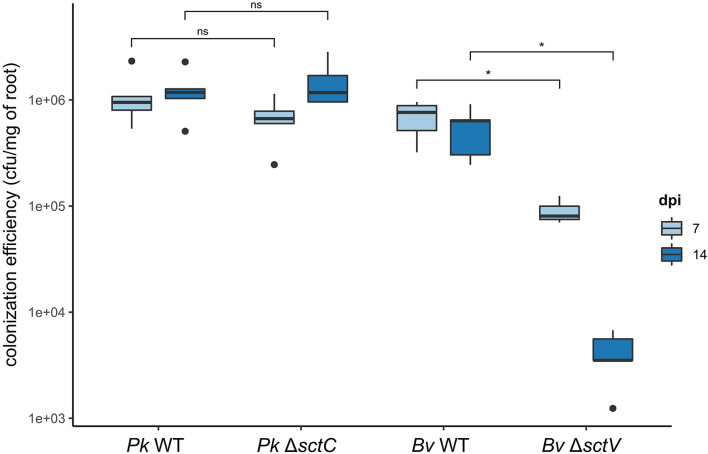
Effect of T3SS loss on root colonization by *B. vietnamiensis* LMG10929 and *P. kururiensis* M130. Rice seedlings were inoculated with either WT or T3SS deficient strains of *P. kururiensis* M130 or *B. vietnamiensis* LMG10929 and the roots were harvested at 7 and 14 dpi, homogenized and the colonizing bacteria population estimated using serial dilutions and spot counting. Cfu/mg of roots were compared between conditions with a Wilcoxon test (*p < 0.01; ns, not significant). Black dots represent outgrouping samples. Mock inoculations were conducted on a control set of plants which showed a complete absence of developing bacteria at both time-points.

We then compared their colonization efficiency to that of WT strains at different time-points (Figure 5). The mutation has no impact on the colonization capacity of rice roots by *Pk* in the first 2 weeks after inoculation. *Bv* however, is strongly affected by the loss of its T3SS. At 7 dpi, the mutant’s colonization capacity is only at 13% of the WT stain’s capacity. At 14 dpi the mutant’s population collapses further to 0.75% compared to the WT strain.

## Discussion

### The Type 3 Secretion System Is a Frequent and Diversified Tool in *Burkholderia s.l.* Species

T3SS are one major driver of virulence in pathogenic strains and are increasingly described to play a central role in several beneficial associations between bacteria and their host. Besides the mammal pathogens associated to the *Burkholderia mallei* complex, almost no attention has been brought to the T3SS of *Burkholderia s.l.* species. These species were described to engage in a multitude of interactions ranging from symbiosis to pathogenicity with a variety of hosts. *Burkholderia s.l.* species present a strong diversity in T3SS types, with some strains possessing up to four T3SS-coding clusters, suggesting a central role for this macromolecular tool in the interaction capacity of *Burkholderia s.l.* strains. Such diversity is however not unique in the bacterial kingdom. Hu et al. (2017) had shown in a large survey that 38 genera carried multiple T3SSs, all having independent evolutionary origins (Hu et al., 2017). Among these 38 genera, *Burkholderia s.l.* has, however, the greatest diversity of T3SS, even though the original sampling did not cover the entire available species diversity. The T3SS plurality could represent a strategy to achieve an ecologic diversity as an adaptation to multiple hosts or to different levels of interaction with a same host. Indeed, several *Burkholderia s.l.* species have a broad host range that can include organisms from different kingdoms. Naturally, the use of a T3SS is by no means the only mechanism allowing an efficient host colonization. Additional secretion systems involved in the export of proteins are also considerably contributing to the success of a bacteria’s interaction with its host (Tseng et al., 2009). Interestingly, several *Burkholderia s.l.* species bearing one or more injectisome-coding regions have only been isolated from environmental settings to date. Thus, there are strong chances that an unknown or not yet discovered eukaryotic partner exists for those strains. For strains that have so far been isolated from a single host but bear several T3SS, alternative hosts could exist.

T3SS types display a complex distribution both between *Burkholderia s.l.* species but also between different strains of a same species, demonstrating how readily these systems can be acquired or lost. The selective pressures forcing these acquisition/loss dynamics could be set by the diversity of hosts these bacteria are set to colonize. Hence, intra-specific host diversity can be suggested by the heterogeneous distribution of T3SS among strains of a same species. Again, we are limited by the available knowledge on *Burkholderia s.l.* strain ecology to confidently interpret the consequences of intra-specific T3SS heterogeneity.

### The Lack of Effector Detection for the Non-canonical Injectisomes May Suggest Another Role Than Secretion

Among the most frequent injectisomes of *Burkholderia s.l.* are the BCI and PSI types. Both have been noted before in individual strains and some of their particularities have already been reported (Tomich et al., 2003; Mitter et al., 2013; Angus et al., 2014) but they had never been subjected to a wide scale characterization. Here, we report the distribution and conservation of the BCI and PSI among *Burkholderia s.l.* species. Both could be involved in non-virulent plant-colonization as suggested by the ecology of the strains these systems are found in. This supposition is corroborated for the BCI by the rice colonization deficiency of a T3SS insertional mutant of *B. vietnamiensis* LMG10929. Whether it is used for adhesion, as support for biofilm formation or to inject effector proteins, remains to be investigated.

If effectors are secreted through the BCI, they are unlike the effectors which have been identified hitherto for the other T3SS types as we could not detect any of them by homology. Their *de novo* prediction based on the detection of T3 secretion signals was also unsuccessful. This could be resulting from the limits of these computational approaches when screening for non-canonical effectors. Still, an alternative role to protein secretion cannot be excluded at this point.

The BCI is abundant in species of the BCC which are known to efficiently colonize the rhizosphere. However, these are not described to be phytopathogens. With *B. vietnamiensis* as leading figure, several BCC members have demonstrated plant growth promotion as they are potent phytohormone producers and nutrient providers (Trân Van et al., 2000; Rojas-Rojas et al., 2018). To date, the most common T3SS of plant interacting bacteria were those of the Hrp-1 and Hrp-2 type as well as the Rhc for rhizobia (Abby and Rocha, 2012).

### *Burkholderia* Cepacia Complex Injectisome and Hrp-2: Contrasted Roles in Plant-Interaction

Here we suggest that the BCI might be an additional major player in plant interaction but for which effectors remain to be investigated. On the other side, and quite unexpectedly, the rice colonization efficiency of the *P. kururiensis* M130 Hrp-2 mutant was not impacted in the first 14 days of interaction. This result was surprising regarding all the literature on this T3SS type reporting its major role in plant-bacteria interactions. Contrarily to *Bv*, Hrp-2 positive *Paraburkholderia* species such as *Pk*, are often bearing a few homologs of effectors from plant pathogens such as *Ralstonia* or *Pseudomonas.* While interpretations of negative results must always be done with care, we can hypothesize that in the specific case of the *Pk*/Nipponbare association, the interaction does not require the use of an injectisome for colonization. We can further suggest that the T3SS plays little or no role in this precocious stage of plant colonization, but that its absence could rather have a major impact in the later stages, particularly during the establishment of endophytic colonization. The absolute functionality of *Pk*’s Hrp-2 would first need to be validated to safely interpret the present results, although it seems very unlikely that a bacterium would conserve a 24 kb genomic region showing no sign of degeneration if it was not functioning.

There is still much to discover on the general implication of T3SS in beneficial interactions and specifically for *Burkholderia s.l.* species which are promising plant growth promoting strains. A focus on the secreted effector proteins, using a combination of *in silico* predictive tools, transcriptomics, and secretome-proteomics, could provide valuable knowledge on the frontier between pathogenicity and mutualism resulting from the type III secretion.

## Data Availability Statement

The data presented in the study are deposited in the NCBI repository, accession numbers PRJEB31911 and PRJEB42536.

## Author Contributions

AW carried out the data extractions and analyses, plant tests and observations, and drafted the manuscript. IR participated in DNA extraction and strain cultures. NB was involved in mutant construction and plant testing. GB and LM participated to the design, coordination of the work, and to the writing of the manuscript. All authors read and approved the final manuscript.

## Conflict of Interest

The authors declare that the research was conducted in the absence of any commercial or financial relationships that could be construed as a potential conflict of interest.

## Publisher’s Note

All claims expressed in this article are solely those of the authors and do not necessarily represent those of their affiliated organizations, or those of the publisher, the editors and the reviewers. Any product that may be evaluated in this article, or claim that may be made by its manufacturer, is not guaranteed or endorsed by the publisher.

## Supplementary Material

Supplementary Figure 1 Synteny conservation within T3SS types across *Burkholderia s.l.* strains.

Supplementary Figure 2 Distribution of BCI and PSI types outside *Burkholderia s.l.* species. **(A)** Concatenated SctC, SctN, SctT and SctV sequences from the PSI and BCI of *P. phytofirmans* PsJN and *B. vietnamiensis* LMG10929, respectively, were used to screen sequence databases for homologies using BLAST and identify the closest similarities in the bacterial kingdom. **(B)** The SctC, SctN, SctT and SctV sequences of the closest hits (in bold) were retrieved, aligned and concatenated resulting in a 1,656 aa alignment. These sequences were used in a phylogenetic reconstruction alongside representatives of nine T3SS types as before (Figure 2).

Supplementary Figure 3 Side by side comparison of a Burkholderia s.l. and a T3SS phylogeny. Nine concatenated coding sequences (*dnaG, ftsZ, glnA, gyrB, pykA, recA, rho, rpoB*, and *secA*) of the 80 T3SS-bearing *Burkholderia s.l.* strains were used to infer the *Burkholderia s.l.* phylogeny using a Bayesian approach. The right tree is a linearized version of Figure 1. Blue arrows on the left phylogeny exemplify the putative acquisition (right arrows) and losses (left arrow) of the PSI T3SS.

Supplementary Table 1 Listing of T3SS types. For each type we provide a selection of genera where the respective T3SS has been described as well as the type of infection or symbiosis it is most often involved in. (^∗^) Specific strains which were used as reference for their T3SS in our phylogenetic analysis.

Supplementary Table 2 List and details of strains used in this study.

Supplementary Table 3 Bacterial strains and plasmids used in this study.

Supplementary Table 4 T3SS repartition within *Burkholderia s.l.* strains.

Supplementary Table 5 Intraspecific diversity of T3SS distribution for a selection of ten species.

Supplementary Table 6 Curated list of candidate effectors in *Burkholderia s.l.* species.
