# Supplementary figures and images for: Genetic Diversity of Type 3 Secretion System in Burkholderia s.l. and Links With Plant Host Adaptation
Source: Front Microbiol. 2021 Oct 20;12:761215. doi: 10.3389/fmicb.2021.761215 (PMC8565462; doi:10.3389/fmicb.2021.761215)

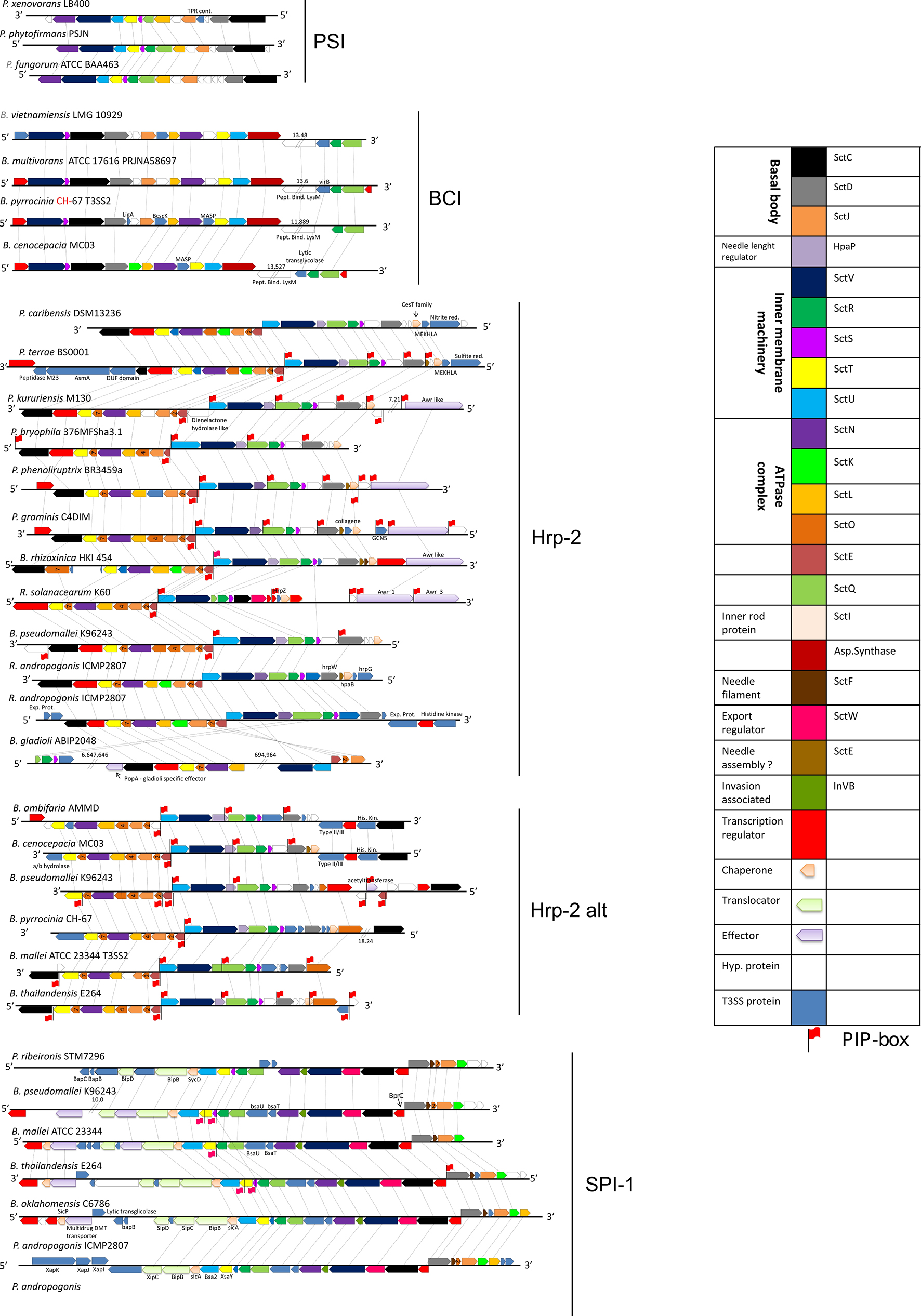

Supplement: Supplementary Figure 1 — Synteny conservation within T3SS types across Burkholderia s.l. strains. [file Image_1.JPEG]

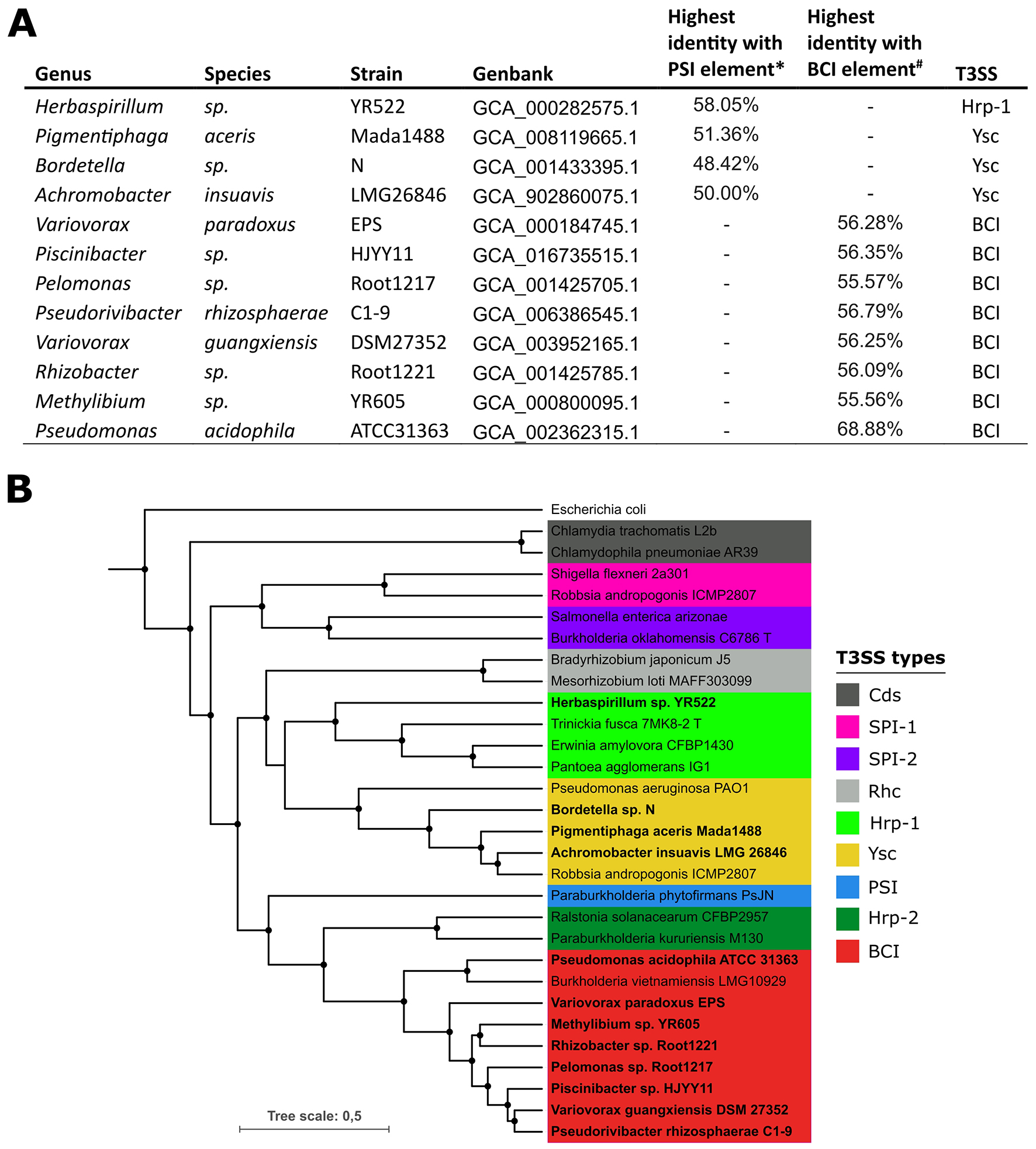

Supplement: Supplementary Figure 2 — Distribution of BCI and PSI types outside Burkholderia s.l. species. (A) Concatenated SctC, SctN, SctT and SctV sequences from the PSI and BCI of P. phytofirmans PsJN and B. vietnamiensis LMG10929, respectively, were used to screen sequence databases for homologies using BLAST and identify the closest similarities in the bacterial kingdom. (B) The SctC, SctN, SctT and SctV sequences of the closest hits (in bold) were retrieved, aligned and concatenated resulting in a 1,656 aa alignment. These sequences were used in a phylogenetic reconstruction alongside representatives of nine T3SS types as before (Figure 2). [file Image_2.JPEG]

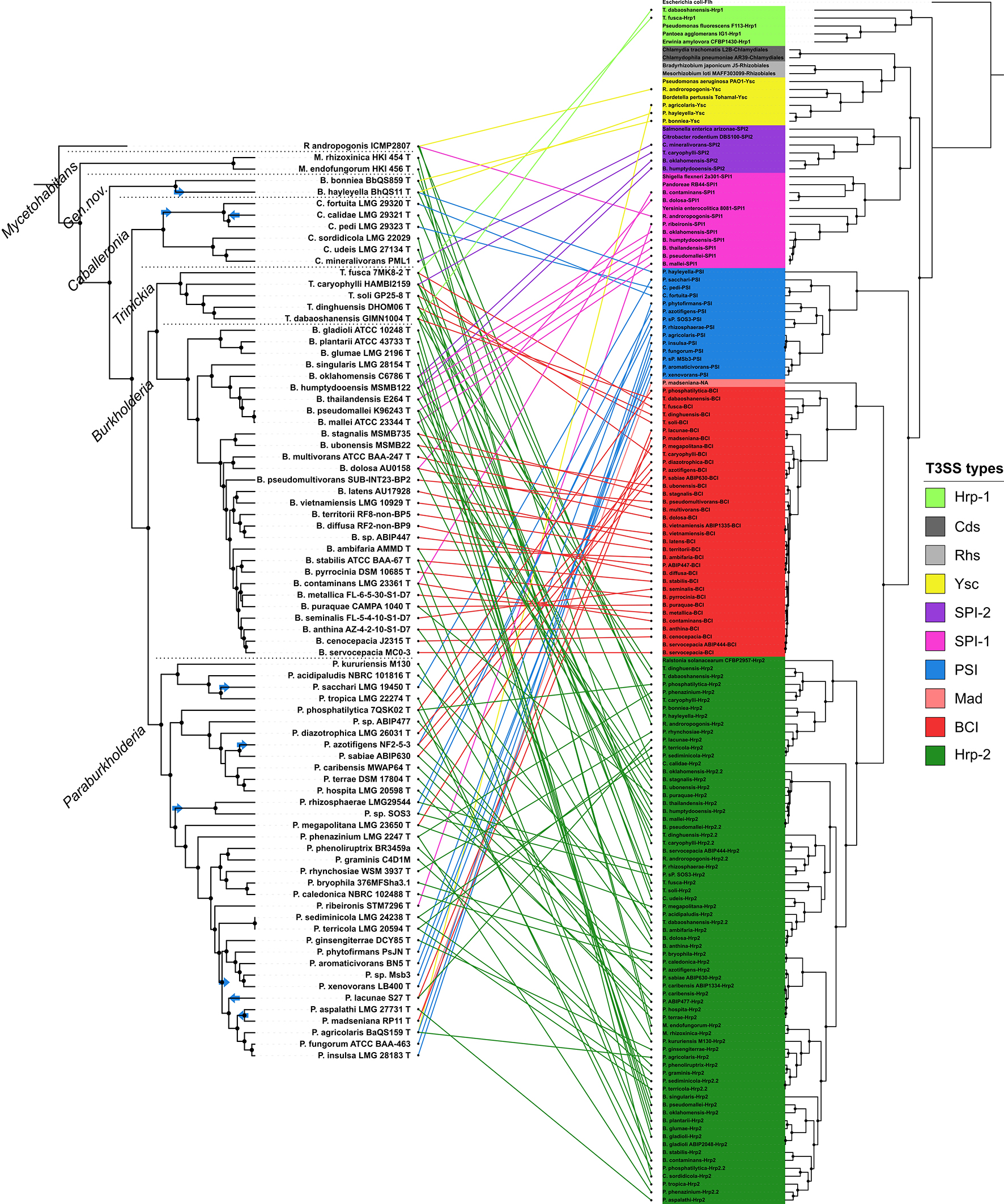

Supplement: Supplementary Figure 3 — Side by side comparison of a Burkholderia s.l. and a T3SS phylogeny. Nine concatenated coding sequences (dnaG, ftsZ, glnA, gyrB, pykA, recA, rho, rpoB, and secA) of the 80 T3SS-bearing Burkholderia s.l. strains were used to infer the Burkholderia s.l. phylogeny using a Bayesian approach. The right tree is a linearized version of Figure 1. Blue arrows on the left phylogeny exemplify the putative acquisition (right arrows) and losses (left arrow) of the PSI T3SS. [file Image_3.JPEG]
