# Supplementary material for: Genetic Diversity of Type 3 Secretion System in Burkholderia s.l. and Links With Plant Host Adaptation
Source: Front Microbiol. 2021 Oct 20;12:761215. doi: 10.3389/fmicb.2021.761215 (PMC8565462; doi:10.3389/fmicb.2021.761215)
Supplement: Supplementary Table 1 — Listing of T3SS types. For each type we provide a selection of genera where the respective T3SS has been described as well as the type of infection or symbiosis it is most often involved in. (∗) Specific strains which were used as reference for their T3SS in our phylogenetic analysis. [file Table_1.DOCX]

Table S1 **Listing of T3SS types.**

| **T3SS type** | **Occurring organisms (non-exhaustive)** | **Ecology involved** |
| --- | --- | --- |
| Hrp-1 | *Pseudomonas fluorescens*F113**, Pantoea agglomerans*IG1**, Erwinia amylovora*CFBP1430* | Plants |
| Hrp-2 | *Xanthomonas, Ralstonia solanacearum*CFBP2957* | Plants |
| Inv/Mxi-Spa (SPI-1) | *Salmonella, Shigella flexneri*2a301**, Yersinia enterolitica*8081* | Humans |
| Ssa-Esc (SPI-2) | Enterohemorrhagic *Escherichia coli*, *Salmonella enterica subsp. arizonae***, Citrobacter rodentium*DBS100*, | Humans |
| Ysc | *Yersinia, Pseudomonas aeruginosa* PA01**, Bordetella pertussis* Tohama I* | Humans |
| Cds | *Chlamydia trachomatis* L2b**, Chlamydophila pneumonia* AR39* | Humans |
| Rhc | *Sinorhizobium, Rhizobium, Cupriavidus*, *Mesorhizobium loti* MAFF303099**, Bradyrhizobium japonicum*J5* | Plants (nodulation) |
| For each type we provide a selection of genera where the respective T3SS has been described as well as the type of infection or symbiosis it is most often involved in. (*) Specific strains which were used as reference for their T3SS in our phylogenetic analysis. | | |
