# Supplementary material for: Genetic Diversity of Type 3 Secretion System in Burkholderia s.l. and Links With Plant Host Adaptation
Source: Front Microbiol. 2021 Oct 20;12:761215. doi: 10.3389/fmicb.2021.761215 (PMC8565462; doi:10.3389/fmicb.2021.761215)
Supplement: Supplementary Table 3 — Bacterial strains and plasmids used in this study. [file Table_3.DOCX]

Table S3 **Bacterial strains and plasmids used in this study.**

| Strains/plasmids | | Relevant characteristics and plasmid constructions | Reference/Source |
| --- | --- | --- | --- |
| ***E. coli*** | |  |  |
|  | JM109 | Δ(*lac-proAB*) | Promega |
|  | DH5α | Δ*recA1*, Δ*endA1*, Δ(*lacZYA-argF*) | ThermoFisher |
|  | CC118 | λpir | (Herrero et al., 1990) |
| ***B. vietnamiensis*** | |  |  |
|  | LMG10929 | Wilde type strain | (Gillis et al., 1995) |
|  | St^R^ | Spontaneous streptomycin resistant clone | This study |
|  | Δ*sctV* | *sctV*::pSHAFT-2 of LMG10929, St^R^, Cm^R^ | This study |
| ***P. kururiensis*** | |  |  |
|  | M130 | Wilde type strain | (Baldani et al., 1997) |
|  | St^R^ | Spontaneous streptomycin resistant clone | This study |
|  | Δ*sctC* | *sctC*::pSHAFT-2 of M130, St^R^, Cm^R^ | This study |
| **Plasmids** | |  |  |
|  | pGEM-T Easy | Cloning vector, Amp^R^ | Promega |
|  | pRK2013 | Helper plasmid for conjugative mobilization of non-self-transmissible plasmids, Kan^R^ | (Knauf and Nester, 1982) |
|  | pSHAFT-2 | Conjugative suicide vector, Cm^R^ | (Shastri et al., 2017) |
|  | pSHAFT-2-*hrcC* | Internal PCR *sctC* fragment of M130 cloned in pSHAFT-2 | This study |
|  | pSHAFT-2-*hrcV* | Internal PCR *sctV* fragment of M130 cloned in pSHAFT-2 | This study |
